# Prognostic Significance of Tumor Growth Rate (TGR) in Patients with Huge Hepatocellular Carcinoma Undergoing Transcatheter Arterial Chemoembolization

**DOI:** 10.3390/curroncol29020038

**Published:** 2022-01-18

**Authors:** Guobin Chen, Xiaoying Xie, Meixia Wang, Xinkun Guo, Zhenzhen Zhang, Lan Zhang, Boheng Zhang

**Affiliations:** 1 Department of Hepatic Oncology, Xiamen Branch, Zhongshan Hospital, Fudan University, Xiamen 361015, China; chen.guobin@zsxmhospital.com (G.C.); xie.xiaoying@zs-hospital.sh.cn (X.X.); wang.meixia@zsxmhospital.com (M.W.); guo.xinkun@zsxmhospital.com (X.G.); zhang.zhenzhen@zsxmhospital.com (Z.Z.); 2 Department of Hepatic Oncology, Zhongshan Hospital, Fudan University, Shanghai 200032, China

**Keywords:** huge hepatocellular carcinoma, tumor growth rate (TGR), transcatheter arterial chemoembolization (TACE), prognosis

## Abstract

The prognostic value of the tumor growth rate (TGR) in huge hepatocellular carcinoma (HHCC) patients treated with transcatheter arterial chemoembolization (TACE) as an initial treatment remains unclear. This two-center retrospective study was conducted in 97 patients suffering from HHCC. Demographic characteristics, oncology characteristics, and some serological markers were collected for analysis. The TGR was significantly linear and associated with the risk of death when applied to restricted cubic splines. The optimal cut-off value of TGR was −8.6%/month, and patients were divided into two groups according to TGR. Kaplan–Meier analysis showed that the high-TGR group had a poorer prognosis. TGR (hazard ratio (HR), 2.06; 95% confidence interval (CI), 1.23–3.43; *p* = 0.006), presence of portal vein tumor thrombus (PVTT) (HR, 1.93; 95% CI, 1.13–3.27; *p* = 0.016), and subsequent combination therapy (HR, 0.59; 95% CI, 0.35–0.99; *p* = 0.047) were independent predictors of OS in the multivariate analysis. The model with TGR was superior to the model without TGR in the DCA analysis. Patients who underwent subsequent combination therapy showed a longer survival in the high-TGR group. This study demonstrated that higher TGR was associated with a worse prognosis in patients with HHCC. These findings will distinguish patients who demand more personalized combination therapy and rigorous surveillance.

## 1. Introduction

Hepatocellular carcinoma (HCC) is a common malignant tumor with high incidence and mortality. Importantly, GLOBOCAN estimated there were 905,000 new cases and 830,000 liver cancer deaths worldwide in 2020 [1]. A tumor nodule larger than 10 cm is defined as huge hepatocellular carcinoma (HHCC). Only some selected patients have a chance of gaining a curative surgery [2,3]. Transcatheter arterial chemoembolization (TACE) is an effective safety treatment to improve the prognosis of HHCC [4,5]. Some patients suffering from unresectable HHCC can switch to resectable tumors by means of preoperative TACE [2].

Presently, there is no distinct prognostic tool to predict the overall survival (OS) of patients with HHCC after TACE. Tumor growth rate (TGR) estimates the dynamic information of the alteration in tumor volume over time (%/month), and has become a metric of progression in many types of tumors, such as adenoid cystic carcinoma, non-small cell lung cancer, renal cell carcinoma, and neuroendocrine tumors [6,7,8,9]. Similarly, TGR might be important in HCC areas. One hundred and eighty-nine advanced HCC patients treated with nivolumab were investigated using TGR [10]. The study revealed that a 40% increase in TGR was the cut-off value used to define hyperprogressive disease (HPD). TGR can serve as a meaningful indicator of HPD induced by anti-PD-1 antibodies. However, there is still a lack of strong evidence to clarify the prognostic value of TGR in HHCC patients treated with TACE.

Therefore, the aim of this study was to explore the prognostic value of TGR variation during early treatment in patients receiving TACE as an initial treatment.

## 2. Methods

### 2.1. Patient Selection

Between January 2018 and October 2020, 97 patients with HHCC in Zhongshan Hospital, Fudan University and Xiamen Branch, undergoing TACE as an initial treatment were retrospectively screened. All patients with primary HCC were diagnosed according to the American Association for the Study of Liver Diseases (AASLD) or European Association for the Study of the Liver (EASL) guidelines. The patients were enrolled based on the following criteria: (1) they had naïve HCC contained nodules with a diameter larger than 10 cm; (2) they underwent TACE as first-line therapy; (3) they had integrated imaging examination of contrast-enhanced CT or enhanced MRI before the initiation of TACE treatment and reexamination in 1 to 3 months postoperatively; (4) they were followed-up from the initial TACE until death or the censor time of the study; (5) their tumors were of the no infiltrative type. Demographic characteristics, oncology characteristics, and some serological markers around the initial TACE were collected for analysis.

### 2.2. TACE Procedure

All TACE procedures were performed by our experienced physicians using traditional interventional radiology. Briefly, after a successful Seldinger puncture in the arteria cruralis, a 5-French catheter selection was carried out to perform arteriography of the superior mesenteric, celiac, and common hepatic arteries. Afterwards, superselective catheterization of the tumor-feeding branch of the hepatic artery was performed with a coaxial microcatheter. After angiography confirmed the location of the catheter, the chemotherapeutic emulsion composed of 10–20 mL lipiodol and 20–40 mg epirubicin was infused into tumor supply vessels. The Embosphere, gelatin sponge, or drug-loaded microspheres were used to strengthen embolism until no tumor staining was observed according to the angiographic results of the HCC.

### 2.3. Follow-Up and TGR Calculation

Living patients were censored on 1 July 2021. Overall survival (OS) was defined as the time from the date of the initial TACE until the death time or the last visit. After treatment with TACE, a follow-up study by repeat contrast-enhanced CT or enhanced MRI was conducted in 1–3 months to judge tumor progression. The assessment was performed by two radiologic physicians using the modification of Response Evaluation Criteria in Solid Tumors criteria (mRECIST). TGR was calculated using a published formula that was previously described [10,11]: TGR = 100 × (exp (TG) − 1); TG (tumor growth) = (3 × log (D2/D1))/time (months), where D1 = tumor size at date 1 when contrast-enhanced CT or enhanced MRI was performed pre-TACE; D2 = tumor size at date 2 when the first imaging examination post-TACE was performed; time (months) = (date 2 − date 1)/30.4; tumor size (D1 and D2) was determined by the longest diameters (SLD) of the largest tumor nodule only.

### 2.4. Statistical Analysis

Continuous variables were described as the mean ± standard deviation and analyzed by independent *t*-tests. Categorical data were described as frequency (percent) and were calculated by using the chi-square test or Fisher’s exact test. We applied restricted cubic splines based on the Cox proportional model to evaluate the dose–response relationship of TGR and the overall survival of patients with HHCC. Time-dependent ROC curve (timeROC) analysis was conducted to evaluate the optimal cut-off value of TGR with the timeROC package in the R-3.6.1 software. The area under the curve (AUC) was calculated. The risk factors for the survival prediction of patients with HHCC were analyzed by Kaplan–Meier survival analysis and Cox regression models. Variables with *p* < 0.05 in the univariate analysis were adjusted as confounders in the multivariate Cox model. The endpoint was overall survival (OS). The mean OS was generated by the means of the Kaplan–Meier curve. Hazard ratios (HRs) and corresponding 95% confidence intervals (CIs) were calculated. The concordance index (c-index) was applied to evaluate model discrimination. Decision curve analysis was conducted to evaluate the clinical utility of the prognostic models.

All analyses were performed using R-3.6.1 software. Two-tailed *p*-values of <0.05 were indicative of statistical significance.

## 3. Results

### 3.1. Optimal Cut-off Value for the TGR

**Figure 1:**
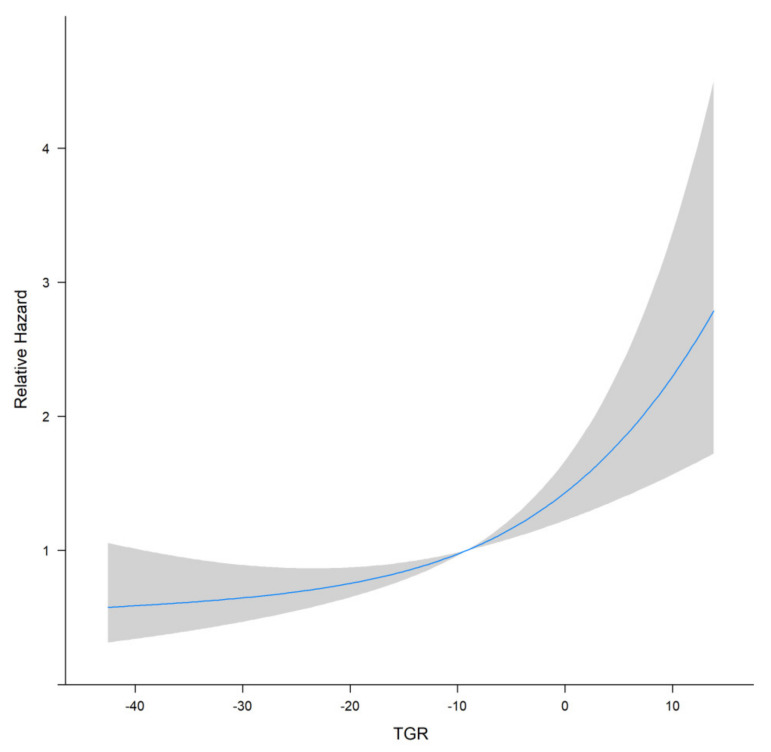
Restricted cubic splines curve of TGR for HHCC patients.

**Figure 2:**
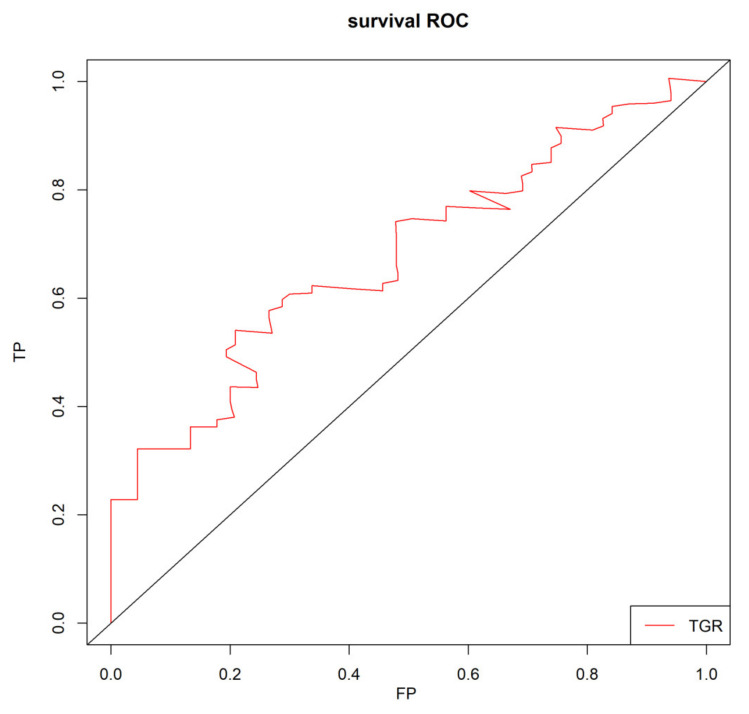
Time-dependent receiver operating characteristic curve (timeROC) for TGR in HHCC patients.

TGR was assessed 1–3 months after the initial TACE procedure. Restricted cubic splines fitted in the Cox proportional hazard model showed that the linear relationship between TGR and overall survival of patients with HHCC (Figure 1, *p* for linear trend <0.001). The greater TGR was associated with the shorter OS of patients with HHCC. Furthermore, the optimal cut-off value was calculated by the timeROC curve (Figure 2). The TGR ideal cut-off value was −8.6%/month. According to the optimal tangent, HHCC patients were divided into high-TGR group (TGR ≥ −8.6%/month) and low-TGR group (TGR < −8.6%/month).

### 3.2. Patients Characteristics and Overall Survival

**Figure 3:**
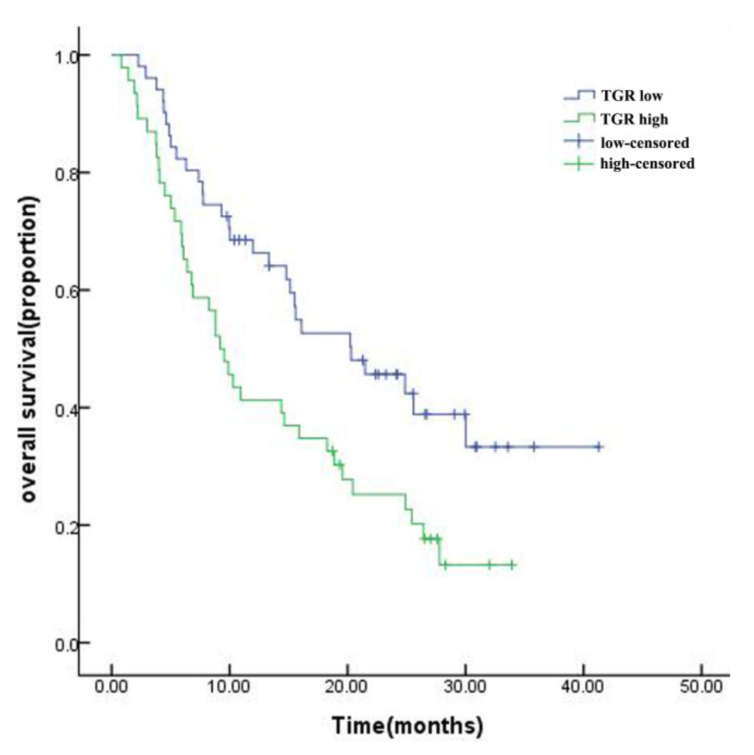
Kaplan–Meier survival curve for HHCC patients in the high-TGR and low-TGR groups.

The baseline characteristics of the study population are presented in Table 1. The median follow-up was 27.6 months, and the median survival of the entire cohort was 14.8 months. The mOS of the low-TGR group was longer than that of the high-TGR group (20.3 vs. 9.2 months, *p* = 0.007, Figure 3). The HHCC cohort included 89 males and 8 females, with an average age of 54.99 ± 11.988 years. The average TGR was −12.52 ± 19.186 and 52.6% for the HHCC patients in the low-TGR group. Hepatitis B virus (HBV) was regarded as the main etiology of HCC, and a greater proportion of HHCC patients (90.7%) suffered from HBV infection. Moreover, 70.1% of the patients had a history of hepatic cirrhosis. The average largest diameter of the tumors was 130.15 ± 24.523 mm, and 56.7% of the patients possessed a single lump. Among them, 51 (52.6%) patients had portal vein thrombosis and some patients (26.8%) had distant metastases. To lessen the interference of collinearity factors, Barcelona Clinic Liver Cancer (BCLC) stage was left out of the additional analyses. More than half of the patients (57.7%) received combination therapy after initial TACE, which included surgery, radiotherapy, targeted therapy, or immunotherapy. The distribution of combination treatments can be found in Supplementary Table S1. In the first radiological evaluation, 11.4% of patients were classified as partial response (PR) and 71.1% of patients were classified as stable disease (SD). However, no patient was evaluated as complete response (CR) (Table 1).

### 3.3. Univariate and Multivariate Analysis

The univariate analysis found that the TGR (*p* = 0.007), the presence of PVTT (PVTT, *p* = 0.033), distant metastases (*p* = 0.027), a lower albumin level (*p* = 0.042), and no subsequent combination therapy (*p* = 0.026) were significantly correlated with OS in patients with HCC (Table 1). Further results of the multivariate analysis are shown in Table 2. Higher TGR (≥−8.6%/month) was an independent prognostic factor for OS (hazard ratio (HR), 2.06; 95% confidence interval (CI) 1.23–3.43, *p* = 0.006). Furthermore, the presence of PVTT (HR, 1.93; 95% CI, 1.13–3.27; *p* = 0.016) was another independent risk factor for OS, and subsequent combination therapy (HR, 0.59; 95% CI, 0.35–0.99; *p* = 0.047) was a protective factor for HHCC patients undergoing initial TACE.

### 3.4. Clinical Value of TGR and Subgroup Analysis

**Figure 4:**
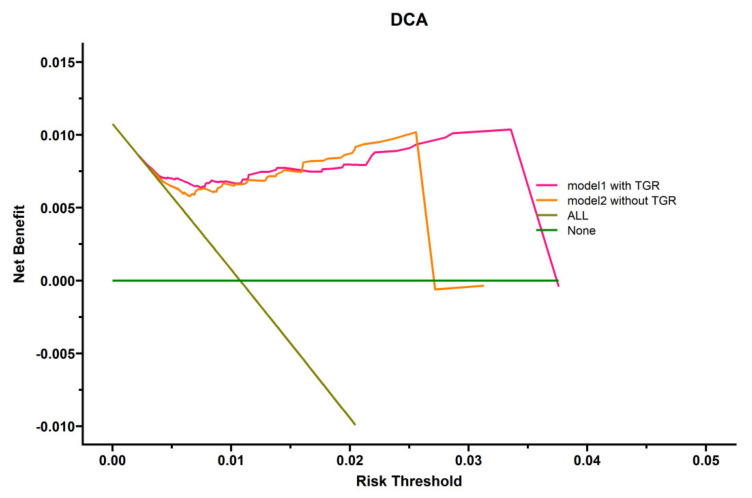
Decision curve analysis (DCA) of several prognostic models in HHCC patients.

To highlight the role of TGR, we explored the clinical utility of the prognostic model by decision curve analysis (DCA). As shown in Figure 4, two models for predicting OS were superior to the all-patient-death scheme or no-patient-death scheme. Furthermore, the model integrating TGR, the presence of PVTT, and with or without subsequent combination therapy in predicting OS was more beneficial than the model without TGR. The model with TGR (c-index 0.700) was better than the model with mRECIST (c-index 0.681).

We further confirmed the relationship between subsequent combination therapy and HHCC patients’ outcomes by analyzing three subgroup cohorts, which were the low-TGR cohort, the high-TGR cohort, and the cohort receiving additional treatment. The HHCC patients with the higher TGR who received the combination therapy after the first TACE showed a longer survival time (mOS 9.9 ± 5.6 vs. 6.9 ± 2.9 months, *p* = 0.038) (Table 3). Nevertheless, the OS of HHCC patients with or without subsequent combination treatment showed no distinct difference in the low-TGR group (*p* = 0.477). As depicted in Table 3, subsequent combination therapy could benefit HHCC patients after initial TACE in the high-TGR group. However, there was no significant difference in survival time among the various application interval times (AITs), which ranged from the initial TACE to the start of the combination therapy.

## 4. Discussion

The early prediction of OS for patients undergoing initial TACE therapy is crucially important for selecting patients who are more likely to benefit from TACE and for optimizing follow-up strategies. To our knowledge, the present study, for the first time, found a linear relationship between the TGR and OS with patients with HHCC who had received TACE as an initial therapy, and higher TGR prompted a poorer prognosis. By means of multivariate analysis, the presence of PVTT and subsequent combination therapy were independent predictors for OS in addition to TGR. In the subgroup analysis, the study showed that combination therapy was more necessary in the high-TGR group.

The HHCC treatment strategy remains controversial on account of these patients frequently having vascular invasion, large tumor size, and multinodular tumor, which have been shown to contribute to a worse survival [12,13,14]. Kim GH et al. showed that the mOS of patients who received TACE as first-line treatment for single, large (>10 cm) HCC was 28 months. In the study, a tumor size of 5–7 cm and grade 1 ALBI seem more suitable for TACE. However, they excluded patients with macrovascular invasion and extrahepatic metastasis [15]. In the current study, the baseline characteristics showed a more aggressive tumor biology in which the largest tumor diameter was 130.15 ± 24.523 mm and more than half of the patients (52.6%) had PVTT. However, there were no befitting methods or tools to predict the OS of patients with HHCC. The predictive role of objective response (OR) by pretreatment TGR in patients with HCC has been reported in a previous study [16]. This study focused on the prediction of treatment efficacy but lacked long-term survival analysis. In another study, objective responses by mRECIST in patients with advanced HCC could well predict OS [17]. However, in our research, it seems to not be associated with prognosis. In addition, the model with TGR was superior to the model with mRECIST. However, this might be due to different baseline status of patients and the influence of TACE treatments not being accounted for. Therefore, a reliable TACE-specific prognostic model is required. In univariate analyses, the hodiernal study extracted higher posttreatment TGR (*p* = 0.007), presence of portal vein tumor thrombus (PVTT, *p* = 0.033), distant metastases (*p* = 0.027), lower albumin level (*p* = 0.042), and no subsequent combination therapy (*p* = 0.026) as potential prognostic factors from demographic characteristics, oncology characteristics, and some serological markers. Afterwards, posttreatment TGR (*p* = 0.006), presence of PVTT (*p* = 0.016), and subsequent combination therapy (*p* = 0.047) were independent predictors of OS when a further multivariate analysis was conducted. The median OS of 20.3 months in the low-TGR cohort was distinctly longer than the 9.2 months in the high-TGR group. Our results demonstrated that posttreatment TGR was linearly correlated with the risk of HHCC, which was an independent predictor of OS. The next DCA also verified the higher clinical value of the combined model with TGR compared with the model without TGR.

Multiple strategies cover single or combination therapies when it comes to the treatment of HCC up to the size and stage of the tumor. The patients with liver resection were associated with longer OS than that of the TACE group [18]. The models based on tumor number, microscopic vascular invasion, tumor differentiation, preoperative alpha-fetoprotein level, albumin–bilirubin grade, liver segment invasion, neutrophil-to-lymphocyte ratio or platelet-to-neutrophil ratio, and surgical margin or intraoperative blood transfusion for predicting the prognosis of HHCC with liver resection showed more accurate prognostic predictions [19]. Unfortunately, a large number of HHCC patients lost the chance to be operated on when they were first diagnosed. TACE can obstruct tumor supply vessels, but has a limited therapeutic effect on HHCC by reason that compactly embolized tumors make it tough to distinguish tumor-feeders on the arteriogram [5]. Combination therapies can be an efficacious approach to intermediate-to-advanced-stage HCC [20]. Palmer et al. reviewed strategies to guide patient selection for locoregional therapies or locoregional–systemic combination therapy. They found significantly better OS results in those receiving the combined treatment than in those receiving TACE alone. A study showed that the one-year survival rate in the ablation plus stereotactic body radiotherapy (SBRT) cohort was 87%, which was overmatched in the SBRT-only group [21]. Another study demonstrated that TACE following percutaneous microwave coagulation therapy in HHCC patients was an ideal treatment strategy. The 6-, 12-, and 18-month OS rates for HHCC patients were 50%, 41.67%, and 16.67% in the combination therapy group [22]. In our study, the HHCC patients with subsequent combination therapy revealed a longer OS in the high-TGR group, which was in accordance with previous research. However, this result could not be obtained in the low-TGR group. Based on the results of this study, the TGR can help us screen out HHCC patients who can benefit from the subsequent combination therapy regardless of the treatment initiation time. In clinical practice, clinicians should recognize patients with higher TGR for subsequent combination therapy, while patients with lower TGR could receive rigorous surveillance. In this way, the economic burden for some HHCC patients can be significantly reduced while ensuring efficacy.

There are, however, some limitations to this study. Firstly, this was a retrospective study, and the number of patients in this study was small. Selection bias is inescapable in observational studies. Secondly, HBV is the main cause of HCC in Chinese patients, especially in HHCC. Moreover, in our study cohort, 90.7% of patients suffered from HBV infection. Some patients with other etiologies such as HCV or alcohol use may exhibit different tumor characteristics. Finally, the use of several kinds of chemotherapeutic drugs and embolization materials when TACE is in operation may interfere with the effectiveness of treatment; e.g., bland transarterial embolization using gelatin sponge particles followed by transarterial chemoembolization using lipiodol mixed with anticancer agents and gelatin sponge particles, which improve survival in patients with HHCC [4]. Further studies are required to establish the standard regime in cTACE for huge HCCs.

## 5. Conclusions

HHCC leaves a short time for treatment, and the selection of appropriate treatment not only improves the curative effect but also reduces the burden of patients. Our study suggests that TGR is an independent risk factor for mortality in HHCC patients undergoing TACE as an initial treatment, and higher-TGR patients may potentially benefit from subsequent combination therapy. The TACE-specific model based on TGR and routinely available clinical features has a higher clinical value. The results of the present study are conducive to the management of patients with huge HCC; however, further validations in larger study populations and in patients with different etiologies remain highly warranted.

## Figures and Tables

**Table 1:** Characteristics and univariate analysis of the HHCC cohort (mean SD/N (%)).

Characteristics	Total	*p*-Value (OS)
Age	55.99 ± 11.988	0.823
Sex		
Male	89 (91.8%)	0.248
Female	8 (8.2%)	
Hepatic Cirrhosis		
No	29 (29.9%)	0.719
Yes	68 (70.1%)	
ECOG performance status		
0	46 (47.4%)	0.065
1	51 (52.6%)	
Diabetes		
No	84 (86.6%)	0.337
Yes	13 (13.4%)	
Hypertension		
No	79 (81.4%)	0.380
Yes	18 (18.6%)	
HBV		
No	9 (9.3%)	0.659
Yes	88 (90.7%)	
Largest tumor diameter (mm)	130.15 ± 24.523	0.429
VI or/and BDI		
No	46 (47.4%)	0.657
Yes	51 (52.6%)	
PVTT		
No	39 (40.2%)	0.033
Yes	58 (59.8%)	
Distant metastases		
No	71 (73.2%)	0.027
Yes	26 (26.8%)	
Tumor number		
Single	55 (56.7%)	0.999
Multiple	42 (43.3%)	
Subsequent combination therapy		
No	41 (42.3%)	0.026
Yes	56 (57.7%)	
Baseline AFP		
≤400	39 (40.2%)	0.735
>400	53 (54.6%)	
Unknown	5 (5.2%)	
Baseline TB (umol/l)	17.56 ± 33.355	0.353
Baseline ALB (g/l)	40.44 ± 5.013	0.042
Baseline ALT (U/L)	50.01 ± 70.696	0.821
Baseline AST (U/L)	84.30 ± 103.765	0.662
Baseline LDH (U/L)	253.58 ± 101.547	0.072
Baseline INR	1.25 ± 1.634	0.051
Baseline NLR	4.12 ± 2.620	0.223
Baseline PLR	190.53 ± 91.734	0.779
Baseline ALBI	−2.66 ± 0.579	0.326
Baseline CRP	29.19 ± 41.321	0.062
Continuous TGR	−12.52 ± 19.186	<0.001
Categorical TGR		
Low (<−8.6)	51 (52.6%)	0.007
High (≥−8.6)	46 (47.4%)	
mRECIST evaluation		
PR	11 (11.4%)	0.071
SD	69 (71.1%))	
PD	17 (17.5%)	

Median with standard deviation is shown for quantitative variables, and counts with proportions are shown for categorical variables. Abbreviations: ECOG—Eastern Cooperative Oncology Group; VI—vascular invasion (without PVTT); BDI—bile duct infringement; PVTT—portal vein tumor thrombosis; AFP—alpha-fetoprotein; TB—total bilirubin; ALB—albumin; ALT—alanine transaminase; AST—aspartate aminotransferase; LDH—lactate dehydrogenase; INR—international normalized ratio; NLR—neutrophil-to-lymphocyte ratio; PLR—platelet/lymphocyte ratio; ALBI—albumin–bilirubin; CRP—c-reactive protein; mRECIST—modified Response Evaluation Criteria in Solid Tumors; PR—partial response; SD—stable disease; PD—progressive disease.

**Table 2:** Multivariate Cox regression analyses for overall survival in HHCC cohort.

Characteristics	HR (95%CI)	*p*-Value
Categorical TGR		
Low (<−8.6)	1.00 (Ref)	0.006
High (≥−8.6)	2.06 (1.23, 3.43)	
PVTT		
No	1.00 (Ref)	0.016
Yes	1.93 (1.13, 3.27)	
Distant metastases		
No	1.00 (Ref)	0.126
Yes	1.51 (0.89, 2.55)	
Subsequent combination therapy		
No	1.00 (Ref)	0.047
Yes	0.59 (0.35, 0.99)	
Baseline ALB (g/l)	0.96 (0.91, 1.00)	0.071

Abbreviations: PVTT—portal vein tumor thrombosis; ALB—albumin.

**Table 3:** Subgroup analysis of subsequent combination therapy (mean SD/N (%)).

Categorical TGR	Subsequent Combination Therapy	Number	mOS	Log-Rank	*p*-Value	AIT	*p*-Value
TGR (<−8.6)	No	18 (35.3%)	15.5 ± 2.7	0.507	0.477		
	Yes	33 (64.7%)	21.5 ± 5.3			2.06 ± 2.74	0.55
TGR (≥−8.6)	No	23 (50%)	6.9 ± 2.9	4.312	0.038		
	Yes	23 (50%)	9.9 ± 5.6			1.83 ± 2.08	0.59

Abbreviations: SD—standard deviation; N—number; AIT—application interval time, which ranged from initial TACE to the start of combination therapy.

## Data Availability

Data from this study are available to researchers who obtain permission from the corresponding author.

## Supplementary Materials

The following supporting information can be downloaded at https://www.mdpi.com/article/10.3390/curroncol29020038/s1, Table S1: The subsequent combination therapy of the HHCC cohort.
