# Supplementary material for: Prognostic Significance of Tumor Growth Rate (TGR) in Patients with Huge Hepatocellular Carcinoma Undergoing Transcatheter Arterial Chemoembolization
Source: Curr Oncol. 2022 Jan 18;29(2):423–32. doi: 10.3390/curroncol29020038 (PMC8870270; doi:10.3390/curroncol29020038)
Supplement: Supplementary file 1 [file curroncol-29-00038-s001.zip › curroncol-1445102-SI.pdf]

Table S1. the subsequent combination therapy of the HHCC cohort.

Treatment	Number (n = 56,%)
Targeted therapy	44 (78.6%)
Immunotherapy	9 (16.1%)
Ablation therapy	3 (5.4%)
Radiation therapy	1 (1.8%)
Surgical resection	10 (17.9%)
